# Low Circulating Monocytes Is in Parallel With Lymphopenia Which Predicts Poor Outcome in Anti-melanoma Differentiation-Associated Gene 5 Antibody-Positive Dermatomyositis-Associated Interstitial Lung Disease

**DOI:** 10.3389/fmed.2021.808875

**Published:** 2022-01-17

**Authors:** Xia Lv, Yuyang Jin, Danting Zhang, Yixuan Li, Yakai Fu, Suli Wang, Yan Ye, Wanlong Wu, Shuang Ye, Bing Yan, Xiaoxiang Chen

**Affiliations:** ^1^Department of Rheumatology, Renji Hospital, School of Medicine, Shanghai Jiaotong University, Shanghai, China; ^2^Department of Rheumatology, West China Hospital, Sichuan University, Chengdu, China

**Keywords:** anti-MDA5 antibody-positive dermatomyositis, monocyte, non-classical monocyte, clinically amyopathic dermatomyositis, prognostic biomarker, rapid progressive interstitial lung disease, lymphocytopenia

## Abstract

Anti-melanoma differentiation-associated gene 5 (MDA5) antibody-positive dermatomyositis (DM)-associated interstitial lung disease (ILD) may progress rapidly and lead to high mortality within 6 or 12 months. Except for reported prognostic factors, simple but powerful prognostic biomarkers are still in need in practice. In this study, we focused on circulating monocyte and lymphocyte counts and their variation tendency in the early stage of ILD. A total of 351 patients from two inception anti-MDA5 antibody-positive cohorts were included in this study, with various treatment choices. Lymphocyte count remained lower in the first month after admission in the non-survivor patients. Although baseline monocyte count showed no significant differences, average monocyte count in the following 4 weeks was also lower in the non-survivor group. Based on the C-index and analysis by the “survminer” R package in the discovery cohort, we chose 0.24 × 109/L as the cutoff value for Mono W0-2, 0.61 × 109/L as the cutoff value for lymph W0-2, and 0.78 × 109/L as the cutoff value for peripheral blood mononuclear cell (PBMC) W0-2, to predict the 6-month all-cause mortality. The Kaplan–Meier survival curves and adjusted hazard ratio with age, gender, and the number of immunosuppressants used all validated that patients with lower average monocyte count, lower average lymphocyte count, or lower average PBMC count in the first 2 weeks after admission had higher 6-month death risk, no matter in the validation cohort or in the pooled data. Furthermore, flow cytometry figured out that non-classical monocytes in patients with anti-MDA5 antibody-positive DM were significantly lower than healthy controls and patients with DM without anti-MDA5 antibodies. In conclusion, this study elucidated the predictive value of monocyte and lymphocyte counts in the early stage and may help rheumatologists to understand the possible pathogenesis of this challenging disease.

## Introduction

Antimelanoma differentiation-associated gene 5 (MDA5) antibody-positive dermatomyositis (DM) is characterized by typical DM rashes, amyopathic or minimal muscle involvement, and rapid progressive interstitial lung disease (RPILD) (1). As a rare but unique subtype of DM without fully understood pathogenesis and limited treatment choice, the mortality within 6 months due to RPILD remains high. Although multiple prognostic factors have been reported in different cohorts, including C-reactive protein (CRP) (2), Krebs von den Lungen-6 (KL-6) (3, 4), serum ferritin (SF) (5, 6), lactate dehydrogenase (LDH) (6), CD4+ CXCR4+ T cells (7), baseline forced vital capacity (FVC) (8), and radiographic features (9), some of the predictors are not universally accessible. Thus, simple but robust prognostic biomarkers are still in need in practice.

According to a previous study (10, 11), complete blood counts analysis revealed that lymphocytopenia was correlated with poor outcomes of clinically amyopathic dermatomyositis (CADM)-associated RPILD. A recent study (11) reported that lower lymphocyte count at baseline was associated with higher mortality in anti-MDA5-associated RPILD. However, with the limited case number, there was no significant difference between baseline lymphocyte count in anti-MDA5 antibody-positive patients with DM with or without ILD. Besides, it is difficult for clinicians to identify the patients who will progress rapidly at diagnosis.

Circulating monocytes, originated from progenitors in the bone marrow and with the potential to differentiate into specific effector cells, such as antigen-presenting cells, macrophages, and fibrocytes, have been implicated in inflammatory and fibrotic diseases (12, 13). In a retrospective, multicenter cohort study (14), high monocyte count (>0.95 × 10^9^/L) was significantly associated with increased risk of all-cause mortality in patients with idiopathic pulmonary fibrosis (IPF) and systemic sclerosis (SSc). Recently, another pooled study further confirmed and tuned down the cutoff value of monocyte count to > 0.6 × 10^9^/L (15). These studies also suggested that monocyte count remained steady during long-term follow-up and was apparently not being affected by treatment.

Therefore, in this study, we focused on circulating lymphocytes and monocytes in anti-MDA5 antibody-positive DM. By using a large inception cohort as the discovery cohort and another retrospective cohort as validation, we aimed at demonstrating the predicted value of circulating lymphocytes and exploring whether circulating monocyte count can predict 6-month all-cause mortality.

## Materials and Methods

### Study Design and Cohorts

The new-onset anti-MDA5 antibody-positive DM inception cohort for hospitalized patients, which served as the discovery cohort, has been described previously (8). Briefly, patients were enrolled from April 2014 to January 2021 in Renji Hospital South Campus. All the patients fulfilled the ENMC 2018 DM classification criteria, with positive anti-MDA5 antibody and ILD confirmed by high-resolution CT (HRCT). A validation cohort retrospectively collected using the same criteria from Renji Hospital West Campus was included. The primary outcome for both the cohorts was 6-month all-cause mortality. This study protocol was approved by the Ethics Committees of Renji hospital and written informed consent was obtained from all the patients.

### Clinical Data Collection

Absolute monocyte count and lymphocyte count were obtained from complete blood counts per week during the first 4 weeks since admission. Absolute monocyte counts were referred as *Mono W0, Mono W1, Mono W2, Mono W3, and Mono W4*; absolute lymphocyte counts were referred as *Lymph W0, Lymph W1, Lymph W2, Lymph W3, and Lymph W4*. Other baseline data, including course of ILD, course of DM, CRP, erythrocyte sedimentation rate (ESR), SF, LDH, maximum creatine kinase (CK), pulmonary function test (PFT) results, were also collected. However, only parameters with <20% missing data in both the cohorts were subjected to further analyses. Treatment information was collected during hospitalization and follow-up, including the maximum dose of glucocorticoids, choice of immunosuppressants, and whether antifibrotic drugs were used.

### Average Monocyte, Lymphocyte, and Peripheral Blood Mononuclear Cell (PBMC) Count Definition

To reduce the sampling variations in a single time point, we evaluated the value of monocyte and lymphocyte weekly in the consecutive 4 weeks from the baseline. The arithmetic mean values of monocyte count were defined as *Mono W0* (the same as absolute monocyte count at baseline), *Mono W0-1* (the mean monocyte count from baseline to week 1)*, Mono W0-2, Mono W0-3*, and *Mono W0-4*. The arithmetic mean values of lymphocyte count were defined in the same way, as *Lymph W0* (the same as absolute lymphocyte count at baseline)*, Lymph W0-1* (the mean lymphocyte count from baseline to week 1)*, Lymph W0-2, Lymph W0-3, and Lymph W0-4*. PBMC was defined as the sum of monocyte and lymphocyte count. We also calculated the arithmetic mean values of PBMC, defined as *PBMC W0, PBMC W0-1, PBMC W0-2, PBMC W0-3*, and *PBMC W0-4*.

### Flow Cytometry

Additional patients with anti-MDA5 antibody-positive DM (*n* = 15) or anti-MDA5 negative DM (*n* = 7), all with ILD, along with 31 healthy controls (HCs) were enrolled during February 2021 and August 2021. Fresh preparation of PBMC was collected by lymphocyte Ficoll. PBMC samples were then stained with the following antibodies within 2 h after being isolated from whole blood and coated with FC-block: Alexa-Floura-430-ZA, APC-cy7-CD3, PE-Texas-Red-CD19, Pacific-blue-HLA-DR, PE-cy7-CD56, FITC-CD88, PERCP-CD89, APC-CD14, and PE-CD16. Classical monocytes were recognized as CD3-CD19-HLA-DR+CD56-CD88+CD89+CD14+CD16-, while non-classical monocytes as CD3-CD19-HLA-DR+CD56-CD88+CD89+CD14loCD16hi and intermediate monocytes as CD3-CD19-HLA-DR+CD56-CD88+CD89+CD14+CD16+. BD Aria II instrument was used for FACS and data were analyzed using Flowjo software version 10 (Tree Star Incorporation, Ashland, Oregon, USA).

### Statistical Analysis

The Statistical Product and Service Solutions(SPSS) version 26.0 was applied in comparing clinical characteristics, cell counts, and correlation analysis. The Mann–Whitney *U* test was applied to compare the mean monocyte count between different groups/cohorts since the Kolmogorov–Smirnov test for normal distribution rejected normality. The Kaplan–Meier estimates were used to obtain the proportion of patients who had an event during follow-up with the GraphPad prism 9. The predictive value of different average cell counts was quantified by the Harrell concordance index (C-index) with 95% CI. Cutoff thresholds of biomarkers were defined based on the “survminer” R package. The Cox proportional hazards model was used to estimate the hazard ratio (HR) and the corresponding two-sided 95% CIs for all-cause mortality. *p-*values <0.05 were considered as statistically significant.

## Results

### Clinical Characteristics of the Discovery and Validation Cohorts

In total, 235 patients admitted to Renji Hospital South Campus were included in the discovery cohort (Table 1), according to the inclusion criteria described above. 62.1% of the patients were female (*n* = 146) and the mean age of this cohort was 49.93 years old (yo). The course of DM/CADM before admission was 3.07 months on average, while the mean course of ILD before admission was 1.48 months (since ILD was diagnosed by HRCT or the respiratory symptoms occurred). In total, 141 patients survived during a 6-month follow-up, suggesting the 6-month all-cause mortality of this discovery cohort was 40%. Immunosuppressants used both before admission and during follow-up were counted and one patient may receive multiple therapeutic regimens. In total, 155 patients (66.0%) received tofacitinib therapy, which reflected the impact of an investigator-initiated clinical study conducted on this campus. Cyclophosphamide, cyclosporin, tacrolimus, rituximab, and mycophenolate mofetil were also used.

**Table 1:** Clinical characteristics of patients in the discovery and validation cohorts.

**Clinical characteristics**	**Discovery cohort** **(*n* = 235)**	**Validation cohort** **(*n* = 116)**	***p* value**
**Baseline data**			
Age (yo)	49.93 ± 11.17 (*n* = 235)	53.11 ± 11.00 (n = 116)	**0.005**
Gender			0.480
Female	146 (62.1%)	77 (66.4%)	
Male	89 (37.9%)	39 (33.6%)	
Course of dermatomyositis (DM)/CADM before admission (mo)	3.07 ± 3.06 (n = 235)	3.58 ± 8.04 (*n* = 116)	0.058
Course of interstitial lung disease before admission (mo)	1.48 ± 0.66 (*n* = 235)	1.45 ± 0.67 (*n* = 116)	0.673
ESR (mm/h)	33.10 ± 22.45 (*n* = 234)	40.43 ± 21.81 (*n* = 116)	**0.002**
CRP (ml/L)	10.34 ± 13.98 (*n* = 235)	11.38 ± 18.86 (*n* = 114)	0.899
Serum ferritin (ng/ml)	1724.87 ± 2536.79 (*n* = 232)	1094.66 ± 1105.34 (*n* = 110)	0.493
Lactate dehydrogenase (U/L)	402.00 ± 280.46 (*n* = 230)	368.57 ± 201.97 (*n* = 113)	0.662
Maximum creatine kinase (U/L)	288.37 ± 570.09 (*n* = 232)	220.08 ± 348.94 (*n* = 115)	0.397
Pneumomediastinum or Pneumothorax	41 (17.4%)	12 (10.3%)	0.084
positive anti-Ro52 antibody	150 (63.8%)	86 (74.1%)	0.054
**Treatment**			
Maximum dosage of methylprednisolone (mg/d)	154.11 ± 136.87 (*n* = 230)	117.91 ± 110.45 (*n* = 116)	**0.001**
Exposure to pulse intravenous methylprednisolone	21 (9.0%)	7 (6.0%)	0.407
Exposure to anti-fibrotic drugs	92 (39.2%)	51 (44.0%)	0.420
Pirfenidone	78 (33.2%)	39 (33.6%)	
Nintedanib	18 (7.7%)	12 (10.3%)	
Exposure to IVIG	112 (47.7%)	60 (51.7%)	0.497
Immunosuppressants			
Tofacitinib	155 (66.0%)	17 (14.7%)	**<0.001**
Cyclophosphamide	54 (23.0%)	25 (21.6%)	0.788
Cyclosporin	47 (20.0%)	32 (27.6%)	0.135
Tacrolimus	37 (15.7%)	55 (47.4%)	**<0.001**
Rituximab	35 (14.9%)	3 (2.6%)	**<0.001**
Mycophenolate mofetil	25 (10.6%)	5 (4.3%)	0.066
**Outcome**			
6-month all-cause death	94 (40.0%)	42 (36.2%)	0.561

*p < 0.05 were in bold*.

We validated in a cohort of 116 patients, who were admitted to Renji Hospital West Campus from November 2017 to February 2021. The average age of patients (53.11 ± 11.00) in the validation cohort was elder than that in the discovery cohort. Gender distributions, as well as the course of DM and ILD before admission, were similar between the two cohorts. The proportion of pneumomediastinum (or pneumothorax) and positive anti-Ro52 antibody were also similar. The validation cohort had lower levels of ESR, while the levels of CRP, maximum CK, SF, and LDH showed no differences. The percentage of patients who received antifibrotic drugs or intravenous immunoglobulins (IVIG) showed no significant differences between the two cohorts either. Patients from the discovery cohort received a higher maximum dosage of methylprednisolone. Notably, calcineurin inhibitors were the first choice in the validation cohort including tacrolimus used in 55 (47.4%) patients and cyclosporin used in 32 (27.6%) patients. The 6-month all-cause mortality in this validation cohort was 36.2%, which showed no significant difference compared with the discovery cohort.

### Lymphocyte and Monocyte Count of the Non-Survivor Group in the First Month Were Significantly Lower

**Figure 1:**
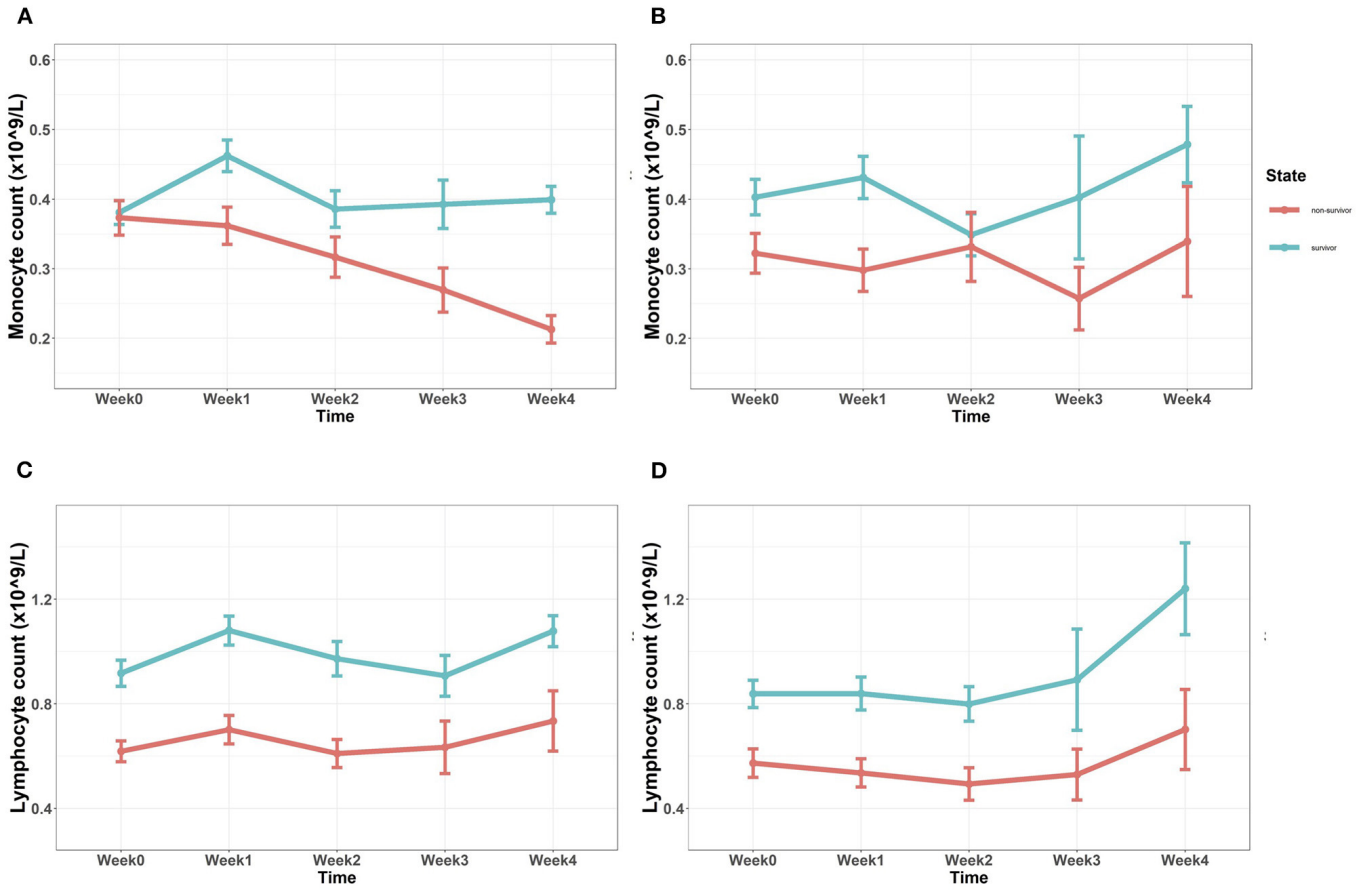
**(A,B)** Tendency of absolute monocyte count after admission in the discovery cohort **(A)** and the validation cohort **(B)**. Baseline monocyte counts showed no significant differences, while monocyte count of the non-survivor group in the subsequent 4 weeks was significantly lower (*p* < 0.05). **(C,D)** Tendency of absolute lymphocyte count after admission in the discovery cohort **(C)** and the validation cohort **(D)**. Absolute lymphocyte count of the non-survivor group in both the cohorts was continuously lower since admission (*p* < 0.05) (Mann–Whitney *U* test).

We collected absolute lymphocyte and monocyte counts from complete blood counts every week in both the cohorts (Figure 1). Absolute and average lymphocyte/monocyte distribution of the two cohorts were first compared (Supplementary Material 1). Monocyte count distribution was similar between two cohorts in these first 4 weeks. Absolute lymphocyte count at 1 week (*Lymph W1*) and average lymphocyte count among the first 2 weeks (*Lymph W0-2*) in the discovery cohort were higher than those in the validation cohort.

Baseline monocyte count (*Mono W0*) showed no significant difference between the 6-month survivor group and the non-survivor group in the discovery cohort (*p* = 0.347); while in the validation cohort, *Mono W0* was a little bit higher in the survivor group, with *p* = 0.040. 7 days later, the monocyte count (*Mono W1*) of the non-survivor group in both the cohorts was significantly lower than that of the survivor group (*p* = 0.001, discovery cohort; *p* = 0.001, validation cohort). The same difference was also observed after 4 weeks (*Mono W4*) (*p* < 0.001, discovery cohort; *p* = 0.048, validation cohort). In the discovery cohort (Figure 1A), monocyte counts of the non-survivor group in 2 weeks (*Mono W2, p* = 0.017) and 3 weeks (*Mono W3, p* = 0.005) were lower than those of the survivor group, while this phenomenon could not be confirmed by the validation cohort (Figure 1B). As shown in Figures 1C,D, baseline lymphocyte counts of the 6-month survivor group were significantly higher than those of the non-survivor group in both the cohorts (*p* < 0.001). In the following 4 weeks, absolute lymphocyte count was continuously lower in the non-survivor group, no matter in which cohort (*p* < 0.001, discovery cohort; *p* < 0.05, validation cohort).

In the discovery cohort, the average monocyte count of the non-survivor group was significantly lower after the first week (*Mono W0-1*), with *p*-value of 0.009 (Supplementary Material 2). Through time, average monocyte count kept lower in the non-survivor group (*p*-value = 0.002, 0.001, <0.001; for *Mono W0-2, Mono W0-3*, and *Mono W0-4*, respectively). The same tendency was observed in the validation cohort. Average monocyte count stayed lower in the non-survivor group than in the survivor group, with *p* = 0.003, 0.005, 0.006, 0.002, for *Mono W0-1, Mono W0-2, Mono W0-3*, and *Mono W0-4*, respectively. Average lymphocyte count of the survivor group, no matter in which cohort, was continuously lower than the survivor group, with *p* < 0.001.

### Average Lymphocyte, Monocyte, and PBMC Count as a Prognostic Biomarker for 6-Month Survival

**Figure 2:**
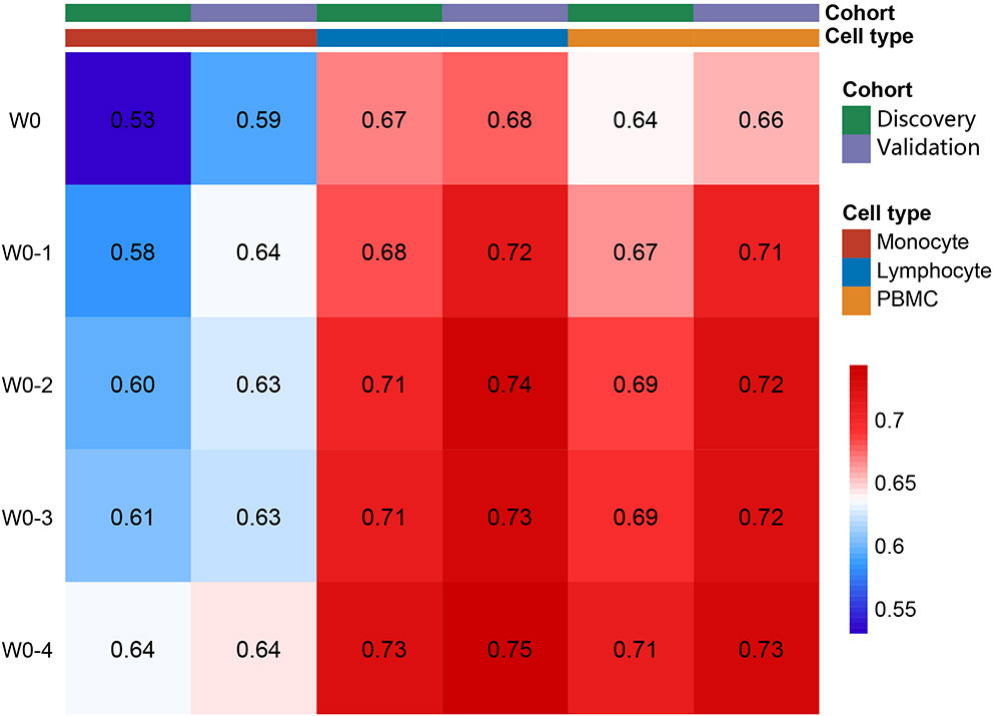
Average lymphocyte, monocyte, and peripheral blood mononuclear cell (PBMC) as risk factors for 6-month mortality. Value in each single block presented the C-index for corresponding biomarkers to predict the mortality.

Average counts of three different cell types (lymphocyte, monocyte, and PBMC) from 5 different time periods (W0, W0-1, W0-2, W0-3, and W0-4) totally formed 15 biomarkers to predict the 6-month mortality in both the cohorts. Risk discriminations of different biomarkers were assessed with C-indexes. As shown in Figure 2, baseline lymphocyte count (*Lymph W0*) and PBMC (*PBMC W0*) showed better prognostic value than baseline monocyte count (*Mono W0*) (ANOVA, *p* < 0.01), both with C-indexes larger than 0.60. Moreover, C-indexes of 3 cell types increased through time in both the cohorts. 2 weeks after admission, C-indexes of average monocyte count reached 0.60 in both the cohorts. Based on this result, average cell counts of three cell types in the first 2 weeks were chosen as biomarkers to predict death in 6 months.

**Figure 3:**
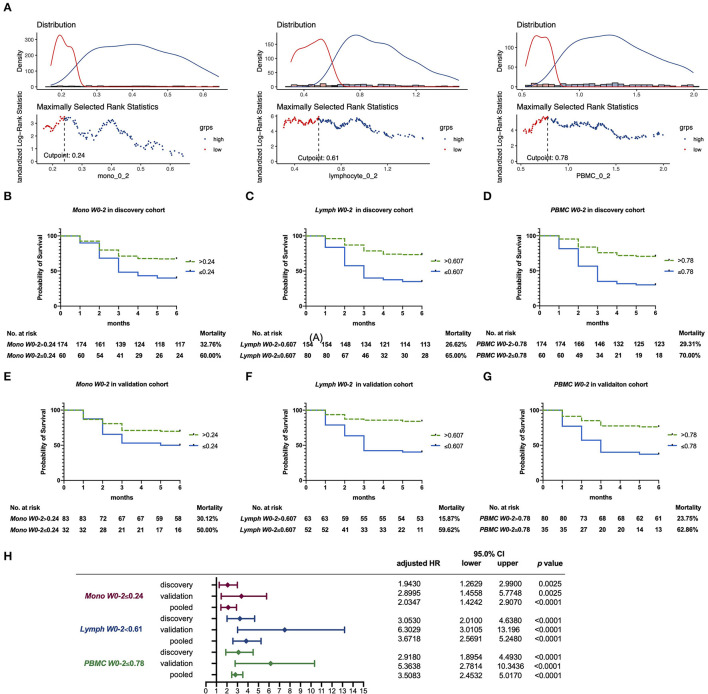
**(A)** Optimal cutoff value for *Lymph W0-2, Mono W0-2*, and *PBMC W0-2* by the “survminer” R package, based on the discovery cohort. **(B–G)** The Kaplan–Meier survival curves displaying 6-month all-cause mortality, based on *Mono W0-2* (with cutoff value of 0.24 × 10^9^/L), *Lymph W0-2* (with cutoff value of 0.61 × 10^9^/L), and *PBMC W0-2* (with cutoff value of 0.78 × 10^9^/L). **(B–D)** The Kaplan–Meier curve in the discovery cohort; **(E–G)** The Kaplan–Meier curve in the validation cohort. **(H)** Adjusted hazard ratio (HR) for average cell counts in two cohorts and in the pooled data, with age, gender, and IS number.

For the convenience of clinical use, optimal cutoff values were used to divide patients into two groups for contrasting mortality. The optimal cutoff values for the chosen biomarkers were derived from the discovery cohort: 0.24 × 10^9^/L for *Mono W0-2*, 0.61 × 10^9^/L for *Lymph W0-2*, and 0.78 × 10^9^/L for *PBMC W0-2* (Figure 3A). The Kaplan–Meier survival curves of the two groups divided by three biomarkers were constructed to show cumulative all-cause mortality over 6-month period (Figures 3B–G). HR of lymphocyte count, monocyte count, and PBMC count were adjusted for age, gender, and IS number. Adjusted HR for average cell counts of three cell types in both the cohorts and in the pooled data was shown in Figure 3H. Patients have divided the 6-month mortality in patients with *Mono W0-2* < 0.24 × 10^9^/L was 60.00%, while mortality of the others was 32.76% (Figure 3B). Mortality of patients with *Lymph W0-2* < 0.61 × 10^9^/L was 65.00%, while others were 26.62% (Figure 3C). The mortality of patients with *PBMC W0-2* < 0.78 × 10^9^/L was 70.00%, while others were 29.31% (Figure 3D). The K–M survival curves also showed significant differences in the validation cohort (Figures 3E–G). These confirm the robustness of our cutoff values.

We also analyzed the relationship between *Mono W0-2*/*Lymph W0-2/PBMC W0-2* and other known prognostic factors in the discovery cohort (Supplementary Material 3). Circulating lymphocyte/monocyte/PBMC in the first 2 weeks were positively correlated to FVC and oxygenation index (OI), while negatively correlated to HRCT score, CRP, ferritin, LDH, and whether pneumomediastinum or pneumothorax occurred, demonstrating that lower *Mono W0-2, Lymph W0-2, and PBMC W0-2* were as credible as previously reported biomarkers.

### Non-Classical Monocytes in PBMCs From Patients With MDA5+DM ILD Were Significantly Lower

**Figure 4:**
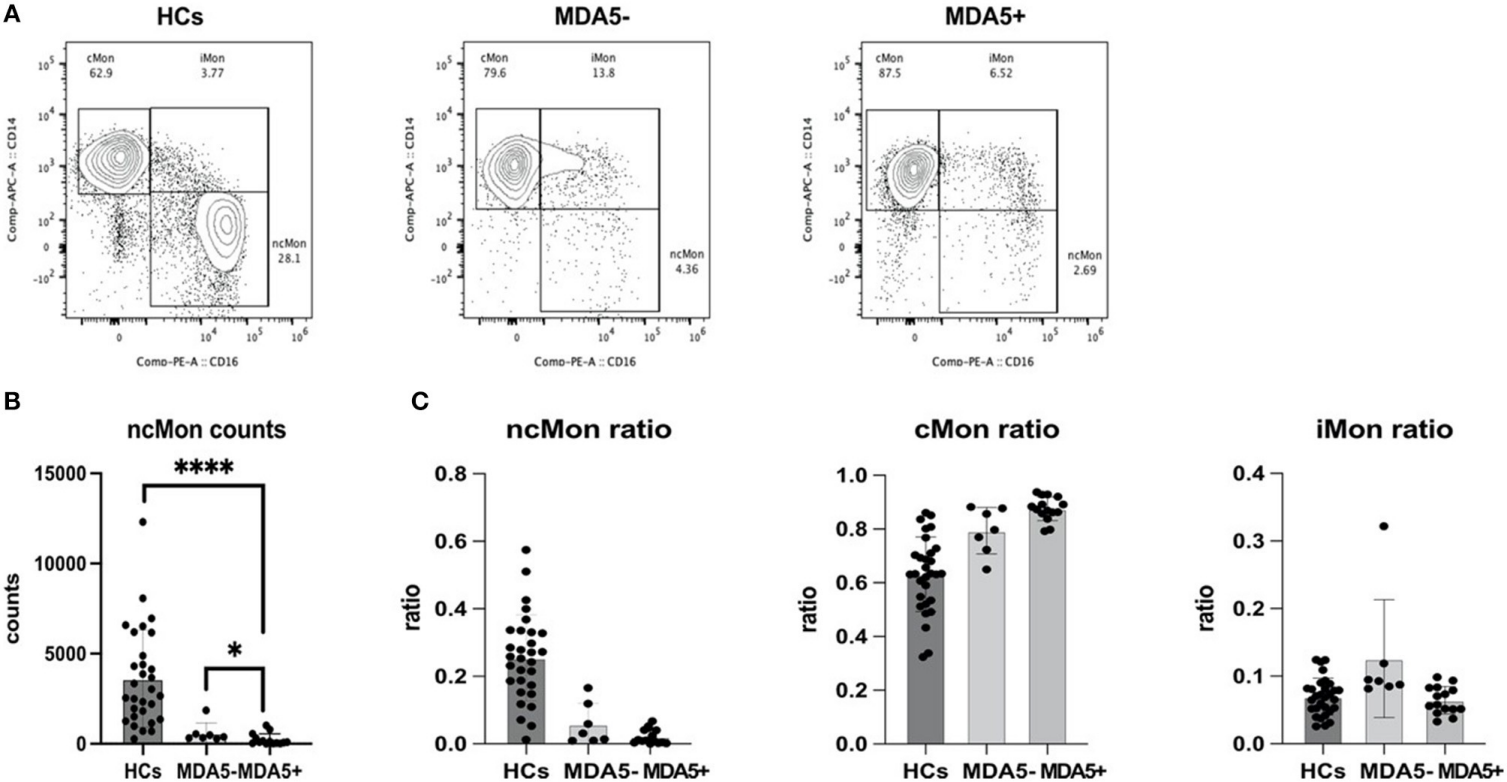
Flow cytometry data demonstrates an excursion of monocyte phenotypes. **(A)** Representative figure of monocyte phenotypes of healthy controls (HCs), patients with anti-MDA5- (MDA5–) and patients with anti-MDA5+ (MDA5+); **(B)** ncMon of patients with MDA5+ significantly decreased compared with HCs (*p* < 0.0001, Mann–Whitney *U* test) and patients with MDA5- (*p* = 0.0250, Mann–Whitney *U* test); **(C)** A significant excursion of ratios of ncMon (*p* < 0.0001, Kruskal–Wallis test), cMon (*p* < 0.0001, Kruskal–Wallis test), and iMon (*p* = 0.0102, Kruskal–Wallis test) occurs among the different groups. *p < 0.05; ****p < 0.0001.

Using FACS, we characterized different PBMC cell types of HCs and patients with MDA5-DM and MDA5+DM. Interestingly, data showed a distinct excursion of monocyte phenotypes (Figure 4A). Although no statistical significance has been found among counts of the total, classical and intermediate monocytes in different groups, the count of non-classical monocytes remarkably decreased in patients with MDA5+DM (Figure 4B). Therefore, the constituent ratio of different monocyte phenotypes showed a significant difference between the different groups (Figure 4C). While monocytes of HCs contain more ncMon and less cMon relatively, monocytes of patients with MDA5+ contain less ncMon and more cMon in their PBMCs.

## Discussion

This retrospective cohort study found that lower monocyte count and lymphocyte count collected from complete blood counts per week in the early course of ILD can predict poor outcomes in anti-MDA5 antibody-positive DM.

At baseline, there was no statistical difference in monocyte count between 6-month survivors and non-survivors. However, within the first 4 weeks after admission, absolute monocyte counts of the 6-month survivors were significantly higher in the discovery cohort. Similar tendency was observed in the validation cohort, though the difference at Week 2 and Week 3 did not reach statistical significance. As with lymphocytes, lymphocytopenia was observed at the base in the non-survivors, and continuously existed in the first 4 weeks. In both cohorts, there was a certain percentage of patients who were discharged or died within 7 days after admission. Besides, only part of patients had a complete blood count every week. To avoid data missing or censor of extreme cases, we used the mean cell counts as prognostic biomarker candidates. Average cell count can also reflect the changing tendency of PBMC and avoid short-term fluctuation due to treatment like intravenous pulse steroids.

Based on this definition, we found that the average lymphocyte and average monocyte count of the 6-month survivor group were significantly higher than those of the non-survivor group, both in the discovery cohort and in the validation cohort. C-index helped to show the predicted value of average cell count, including average PBMC count in 6-month survival. All three kinds of average cell count predict better through time. Considering the clinical need in accessing disease activity and prognosis early and promptly, we chose Week 2 after admission as a considerable time point. *Mono W0-2* < 0.24 × 10^9^/L, *Lymph W0-2* < 0.61 × 10^9^/L, and *PBMC W0-2* < 0.78 × 10^9^/L could help to identify patients who were at higher risk of death within 6 months. Adjusted HR with age, gender, and the number of immunosuppressants used in both cohorts and in the pooled data were all significantly larger than 1.0, demonstrating that low circulating monocyte, as well as lymphocytopenia, were robust prognostic factors for 6-month all-cause death. Absolute lymphocyte and monocyte counts from complete blood counts (CBC) were easy to be obtained and repeated in the clinic, compared to other clinical data like PFTs. By accessing the PBMC count weekly, the result of our study may help rheumatologists to predict the prognosis of anti-MDA5 antibody-positive patients with DM in the early stage of disease, especially soon after ILD onset.

Patients with lymphocytopenia had a higher mortality risk in this study, which validated the result of a recent cohort study mentioned above (11). Lymphocyte counts were thought to be sensitive to glucocorticoid and immunosuppressive treatment, while lymphocytopenia was consistent in the non-survivor group in both discovery and validation cohort in this study.

An early study reported that patients with DM can have lymphocytopenia, manifested as lower peripheral blood CD4+ and CD8+ T-cell and B-cell absolute counts before the initial treatment, and significant increase in lymphocyte count after treatment (16). Another research further reported that the decreased number of CD3+ T cells and the decreased percentage of CD3+CD4+ T cells were correlated with the presence of ILD in PM/DM (17). Recent research on anti-MDA5+ DM revealed lymphocytopenia in circulation before treatment, and CD8+ T cells decreased further in cases with exacerbated lung interstitial lesions after treatment (18). There can be several explanations for the decrease of lymphocytes in patients with DM. Shu et al. revealed a suppressed autophagy function in T cell of patients with DM, leading to upregulation of T-lymphocyte apoptosis and lymphocytopenia (19). Lymphocytopenia was also reported in virus infections such as coronavirus disease 2019 (COVID-19) (20), which were possibly correlated with reduced lymphopoiesis and excited lymphocytes cell death due to hyperproinflammatory cytokines (21). Acute respiratory distress syndrome featured by cytokine storm syndrome is also one of the main causes of early deaths in patients with RPILD with positive anti-MDA5 antibodies. Among the explosively increased cytokines, type 1 interferon has been accepted as the anchor one (22). It has been demonstrated that increased type 1 interferon can downregulate central memory and naïve T lymphocytes, activate B lymphocytes, and eventually cause lymphocytopenia (23, 24). The possibility also exists that lymphocytes in circulation are activated, recruited to target organs such as lungs and thus consumed after being activated by cytokines. Bronchoalveolar lavage fluid (BALF) from patients with CADM-ILD, with higher possibility to develop RPILD, was reported to have significantly higher lymphocyte ratio than BALF from patients with classic DM-ILD (25). Overall, further research is still needed to demonstrate the detailed mechanism of lymphocytopenia in anti-MDA5 antibody-positive patients with DM-ILD.

Although the first monocyte count cannot be used to predict the prognosis precisely, patients with remarkably low monocyte count (<0.24 × 10^9^/L) at baseline and consistently low monocyte count in the following 2 weeks may have higher 6-month all-cause mortality. This result seems to be not consistent with previous research in IPF or SSc. As mentioned above, circulating monocyte count was thought to be negatively related to the outcome in patients with IPF (14, 15, 26). In another Japanese research in acute exacerbation of fibrosing ILD, absolute monocyte count of more than 0.38 × 10^9^/L was a significant risk factor (27). Interestingly, though there has not been reported on total circulation monocyte decrease in fibrosing ILD, non-classical and intermediate monocyte has also been observed decreasing in circulation and reported migrating to lungs in fibrosing ILD, indicating that quite a part of circulation monocyte is chemotactic (28, 29). The reason for this difference could be complex. ILD progression in patients with IPF generally developed much slower than in patients with anti-MDA5 antibody-positive DM/CADM. The invariability or increase of absolute monocyte count may be ascribed to a compensatory increase of classical monocyte in a long course of the disease. Notably, flow cytometry results in our study also demonstrated the decrease of non-classical monocyte in patients with anti-MDA5 positive DM. Similar phenomenon was observed in patients with COVID-19 with acute lung injury that circulating non-classical and intermediate monocytes were decreased, while monocyte-induced cytokine storm was observed (30, 31). We presume the migratory process may be alike in different diseases but the differential process of monocyte in lung tissue can be diverse. The monocyte function in the pathogenesis of these two kinds of ILD could be different. Although fibroblasts derived from monocytes played a critical role in the progression of IPF (13), acute inflammation through type 1 interferon (IFN) pathway and activated monocytes/macrophages could be responsible for anti-MDA5-associated RPILD (32, 33). However, the exact cause of this phenomenon and the immunological mechanism underlying requires further research. Besides, the primary outcome in this study was all-cause mortality, including deaths due to infections. With lower monocyte count and the usage of glucocorticoids and immunosuppressants, the normal innate immune response function (13) in patients with anti-MDA5 antibody-positive DM may be injured, leading to severe or even fatal infections.

This study has several limitations. The biggest limitation of this study is its retrospective nature, leading to data missing in the validation cohort. Although the two cohorts included in this study are among the largest inception anti-MDA5 antibody-positive DM cohorts even in the world, the relatively small number of patients included may bring unavoidable bias. Simple flow cytometry result has figured out that the subsets of circulating non-classical monocyte decreased in patients with anti-MDA5 antibody-positive DM; however, the sample size of flow cytometry is relatively limited. Further research should be conducted aiming at answering the following questions: Why are the non-classical monocytes in patients with anti-MDA5 antibody-positive DM lower than others? Where are the circulating monocytes going? How do they function in the injured tissue?

In conclusion, this study provides evidence of a novel and simple prognostic biomarker in anti-MDA5 antibody-positive DM/CADM. Future research is required to demonstrate the pathogenic role of monocytes and the mechanism of lymphocytopenia in this disease and larger cohorts are also required to validate the predictive effect of early PBMC count.

## Data Availability Statement

The raw data supporting the conclusions of this article will be made available by the authors, without undue reservation.

## Ethics Statement

The studies involving human participants were reviewed and approved by the Ethics Committees of Renji Hospital. The patients/participants provided their written informed consent to participate in this study.

## Author Contributions

XC, BY, and SY contributed to the conception and design of the study. XL, YY, and WW organized the database. XL and DZ performed the statistical analysis. YJ, YL, and YF performed the flow cytometry. XL wrote the first draft of the manuscript. YJ, DZ, and SW wrote sections of the manuscript. All authors contributed to manuscript revision, read, and approved the submitted version.

## Funding

This study was supported by grants from the National Natural Science Foundation of China (No. 81172870).

## Conflict of Interest

The authors declare that the research was conducted in the absence of any commercial or financial relationships that could be construed as a potential conflict of interest.

## Publisher's Note

All claims expressed in this article are solely those of the authors and do not necessarily represent those of their affiliated organizations, or those of the publisher, the editors and the reviewers. Any product that may be evaluated in this article, or claim that may be made by its manufacturer, is not guaranteed or endorsed by the publisher.
