# Supplementary material for: Low Circulating Monocytes Is in Parallel With Lymphopenia Which Predicts Poor Outcome in Anti-melanoma Differentiation-Associated Gene 5 Antibody-Positive Dermatomyositis-Associated Interstitial Lung Disease
Source: Front Med (Lausanne). 2022 Jan 17;8:808875. doi: 10.3389/fmed.2021.808875 (PMC8802832; doi:10.3389/fmed.2021.808875)
Supplement: Supplementary file 1 [file Data_Sheet_1.pdf]

**Supplementary Material 1. Comparison of absolute and average lymphocyte and monocyte count between two cohorts. Mann-Whitney test.**

| | discovery cohort | n | validation cohort | n | *p* value |
|---|---|---|---|---|---|
| **Absolute lymphocyte count (x10$^9$/L)** | | | | | |
| *Lymph W0* | 0.7975±0.5410 (0.09-4.37) | 235 | 0.7437±0.4340 (0.06-2.40) | 115 | 0.523 |
| *Lymph W1* | 0.9213±0.5858 (0.13-2.77) | 198 | 0.7205±0.4456 (0.10-3.04) | 92 | **0.009** |
| *Lymph W2* | 0.8092±0.5789 (0.05-3.36) | 160 | 0.6763±0.4227 (0.09-1.95) | 72 | 0.151 |
| *Lymph W3* | 0.7907±0.6548 (0.07-3.54) | 108 | 0.7235±0.7562 (0.16-4.66) | 43 | 0.380 |
| *Lymph W4* | 0.9809±0.6319 (0.05-3.38) | 131 | 1.016±0.8717 (0.16-4.46) | 48 | 0.529 |
| **Absolute monocyte count (x10$^9$/L)** | | | | | |
| *Mono W0* | 0.3779±0.2177 (0.00-1.08) | 234 | 0.3743±0.2099 (0.03-1.08) | 115 | 0.893 |
| *Mono W1* | 0.4202±0.2479 (0.02-1.28) | 198 | 0.3791±0.2194 (0.04-1.12) | 92 | 0.205 |
| *Mono W2* | 0.3549±0.2476 (0.03-1.48) | 160 | 0.3421±0.2264 (0.04-1.26) | 72 | 0.771 |
| *Mono W3* | 0.3403±0.2567 (0.00-1.73) | 108 | 0.3351±0.3420 (0.03-2.07) | 43 | 0.385 |
| *Mono W4* | 0.3466±0.1914 (0.03-0.91) | 131 | 0.4206±0.3218 (0.02-1.40) | 48 | 0.588 |
| **Average lymphocyte count (x10$^9$/L)** | | | | | |
| *Lymph W0-1* | 0.8483±0.4872 (0.090-3.260) | 235 | 0.7435±0.3882 (0.155-2.720) | 115 | 0.062 |
| *Lymph W0-2* | 0.8474±0.4852 (0.113-3.260) | 235 | 0.7401±0.3927 (0.133-2.720) | 116 | **0.047** |
| *Lymph W0-3* | 0.8484±0.4937 (0.113-3.260) | 235 | 0.7484±0.4309 (0.133-3.367) | 116 | 0.060 |
| *Lymph W0-4* | 0.8602±0.4794 (0.113-2.633) | 235 | 0.7834±0.4443 (0.133-3.367) | 116 | 0.137 |
| **Average monocyte count (x10$^9$/L)** | | | | | |
| *Mono W0-1* | 0.3982±0.2001 (0.020-1.280) | 235 | 0.3767±0.1857 (0.040-1.120) | 115 | 0.387 |
| *Mono W0-2* | 0.3877±0.1904 (0.045-1.070) | 235 | 0.3727±0.1829 (0.080-0.913) | 116 | 0.476 |
| *Mono W0-3* | 0.3805±0.1877 (0.045-1.070) | 235 | 0.3770±0.2034 (0.088-1.340) | 116 | 0.652 |
| *Mono W0-4* | 0.3770±0.1781 (0.045-1.070) | 235 | 0.3834±0.2076 (0.098-1.340) | 116 | 0.829 |

**Supplementary Material 2. Average lymphocyte and monocyte count of both cohorts at baseline and during first four weeks after admission. Mann-Whitney test.**

| | **discovery cohort** | | | **validation cohort** | | |
|---|---|---|---|---|---|---|
| | non-survivor | survivor | *p* value | non-survivor | survivor | *p* value |
| ***Lymph W0*** | 0.618±0.383 | 0.917±0.597 | <0.001 | 0.573±0.348 | 0.838±0.450 | <0.001 |
| ***Lymph W0-1*** | 0.651±0.361 | 0.980±0.516 | <0.001 | 0.560±0.300 | 0.845±0.396 | <0.001 |
| ***Lymph W0-2*** | 0.626±0.350 | 0.995±0.507 | <0.001 | 0.544±0.293 | 0.851±0.400 | <0.001 |
| ***Lymph W0-3*** | 0.622±0.364 | 1.000±0.512 | <0.001 | 0.546±0.306 | 0.863±0.451 | <0.001 |
| ***Lymph W0-4*** | 0.626±0.378 | 1.017±0.477 | <0.001 | 0.557±0.305 | 0.912±0.461 | <0.001 |
| ***Mono W0*** | 0.373±0.239 | 0.381±0.203 | 0.347 | 0.322±0.184 | 0.403±0.219 | 0.040 |
| ***Mono W0-1*** | 0.368±0.214 | 0.418±0.188 | 0.009 | 0.307±0.150 | 0.415±0.193 | 0.003 |
| ***Mono W0-2*** | 0.348±0.197 | 0.414±0.182 | 0.002 | 0.313±0.165 | 0.407±0.185 | 0.005 |
| ***Mono W0-3*** | 0.335±0.191 | 0.410±0.180 | 0.001 | 0.311±0.166 | 0.414±0.214 | 0.006 |
| ***Mono W0-4*** | 0.323±0.181 | 0.413±0.167 | <0.001 | 0.310±0.164 | 0.425±0.219 | 0.002 |

**Supplementary Material 3. Relationship between average lymphocytes/monocytes/PBMC in the first two weeks and other prognostic factors in the discovery cohort.** ***p*****<0.05, Pearson analysis and Spearman analysis.**

| | ***Mono W0-2*** | | ***Lymph W0-2*** | | ***PBMC W0-2*** | |
|---|---|---|---|---|---|---|
| | r | Sig. | r | Sig. | r | Sig. |
| **FVC (n=186)** | 0.109 | 0.139 | 0.219 | **0.003** | 0.211 | **0.004** |
| **OI (n=235)*** | 0.246 | **<0.001** | 0.413 | **<0.001** | 0.407 | **<0.001** |
| **HRCT score (n=221)** | -0.252 | **<0.001** | -0.416 | **<0.001** | -0.413 | **<0.001** |
| **CRP (n=235)** | -0.172 | **0.008** | -0.123 | 0.059 | -0.152 | **0.019** |
| **SF (n=232)** | -0.080 | 0.223 | -0.197 | **0.003** | -0.183 | **0.005** |
| **LDH (n=230)** | -0.287 | **<0.001** | -0.322 | **<0.001** | -0.348 | **<0.001** |
| **Ulcers (n=235)** | -0.021 | 0.751 | -0.043 | 0.515 | -0.028 | 0.670 |
| **Arthritis (n=235)** | -0.018 | 0.787 | 0.045 | 0.491 | 0.014 | 0.827 |
| **Muscular involvement (n=235)** | -0.016 | 0.805 | -0.014 | 0.829 | -0.018 | 0.789 |
| **Pneumomediastinum /Pneumothorax (n=235)** | -0.130 | **0.046** | -0.231 | **<0.001** | -0.213 | **0.001** |

*OI, oxygenation index.
